# Supplementary material for: COVID-19 Preparedness and Perceived Safety in Nursing Homes in Southern Portugal: A Cross-Sectional Survey-Based Study in the Initial Phases of the Pandemic
Source: Int J Environ Res Public Health. 2021 Jul 28;18(15):7983. doi: 10.3390/ijerph18157983 (PMC8345424; doi:10.3390/ijerph18157983)
Supplement: Supplementary file 1 [file ijerph-18-07983-s001.zip › File S1.pdf]

Supplementary File 1

Nursing home COVID-19 preparedness checklist

1. Characterization of the Institution						
Name						
Respondent's name				Date		
No. bedrooms		Laundry		No. interior leisure space		
		Rest areas for personnel		No. outdoor leisure space		

Residents						
	Total	Autonomous	Bedridden	In wheelchair	Reduced mobility	How many could be hosted by family members?
No. men						
No. women						
No. positive for COVID-19:	No. prophylactic isolation:	Reason(s):				

Personnel				
	Total	Positive for COVID-19	In prophylactic isolation	Reason(s)
Internal				
External				
Brief description of absenteeism among internal professionals and implications to the contingency plan				

2. Structure for planning and decision making		Completed	In progress	Not initiated
Response to COVID-19 has been incorporated into emergency management planning for the facility.		☐	☐	☐
Date of last update:				
A multidisciplinary planning team has been created to specifically address COVID-19 preparedness planning and monitoring.		☐	☐	☐
A COVID-19 response coordinator has been assigned.		☐	☐	☐
Identify this professional	Name			
	Telephone			
	Email			
	Educational background:			
Notes				

3. COVID-19 contingency plan	Completed	In progress	Not initiated
There is a contingency plan in place that introduces mechanisms to protect the health of users, professionals, and potential visitors.	☐	☐	☐
A copy of the COVID-19 contingency plan is easily available at the facility and accessible by personnel.	☐	☐	☐
A COVID-19 information monitoring mechanism is available for continuously update of the contingency plan (for example, guidelines from the Directorate-General of Health).	☐	☐	☐
The plan identifies the person(s) authorized to implement the plan and the management, monitoring and evaluation structure that will be used.	☐	☐	☐
Notes			

4. Elements of a COVID-19 plan		Completed	In progress	Not initiated
**4.1. General**				
A person has been assigned responsibility for monitoring information set forth by the competent public health authorities and updating the COVID-19 response coordinator in the facility.		☐	☐	☐
Identify this professional	Name			
	Telephone			
	Email			
The facility has a process for inter-facility transfers that includes notifying personnel and receiving facilities about a resident's suspected or confirmed diagnosis.		☐	☐	☐
The facility has a system for monitoring the evolution of COVID-19 among residents and personnel in the facility. Information from this monitoring system is used to implement prevention interventions.		☐	☐	☐
The facility has infection control policies that outline the precautions recommended to residents and personnel, including the dissemination of information and use of specific signs (e.g., posters and leaflets).		☐	☐	☐
A mechanism for updating the COVID-19 response plan is well defined, including any deadlines.		☐	☐	☐
Notes				
**4.2. Outbreak capacity**				
A contingency personnel plan has been developed that identifies the minimum personnel needs and prioritizes essential services based on residents' characteristics (e.g., functional limitations).		☐	☐	☐

4. Elements of a COVID-19 plan		Completed	In progress	Not initiated
A person has been assigned responsibility for a daily assessment and monitoring of personnel status and needs during a COVID-19 outbreak.		☐	☐	☐
Identify this professional	Name			
	Telephone			
	Email			
Estimates have been made of the quantities of materials and equipment that would be needed during a minimum four-week outbreak.		☐	☐	☐
A rationing strategy has been developed for Personal Protective Equipment (PPE), medicines and other scarce materials.		☐	☐	☐
A contingency plan has been developed for managing an increased need for postmortem care and disposition of deceased residents.		☐	☐	☐
An area in the facility that could be used as a temporary morgue has been identified.		☐	☐	☐
Any plan for expanding the morgue capacity plan been discussed and decided with the competent authorities.		☐	☐	☐
Notes				
**4.3. Communication**				
Key public health points of contact during the stages of the COVID-19 pandemic have been identified.		☐	☐	☐
Communication plans include how visitors should be informed about the importance of monitoring symptoms for 14 days after visiting the facility.		☐	☐	☐
A person has been assigned responsibility for communications with personnel, residents and their families regarding the status, preparedness, and impact of COVID-19 in the facility.		☐	☐	☐
Contact information and communication channels for family members of facility residents are up to date and fully disseminated.		☐	☐	☐
Communication plans include how signs and other methods of communication will be used across the facility.		☐	☐	☐
Notes				
**4.4. Supplies and resources**				
A written and detailed plan for regular cleaning and disinfection of the facility has been developed.		☐	☐	☐
The cleaning and disinfection of the facility is ensured by a specialized company whenever confirmed cases are reported.		☐	☐	☐
The cleaning and disinfection products used at the facility adhere to recommended standards.		☐	☐	☐
Products in use	70% alcohol	☐	Sodium hypochlorite (bleach)	☐

4. Elements of a COVID-19 plan		Completed	In progress	Not initiated
	Hydrogen peroxide	☐	List others, if any	
All cleaning and disinfection products in use at the facility are well described in the cleaning and disinfection plan, and they are properly labeled, packaged, and stored.		☐	☐	☐
The facility has access to disinfectant that is suitable for hospital use for cleaning the surfaces and equipments.		☐	☐	☐
A regular cleaning and disinfection plan for bedpans, urinals and toilet bowls for residents with suspected or confirmed infection has been included in the plan.		☐	☐	☐
The cleaning and disinfection of the most frequently used surfaces (e.g., tables and chairs) has been included in the plan.		☐	☐	☐
The cleaning and disinfection of shared equipment (e.g., thermometers) has been included in the plan.		☐	☐	☐
The cleaning and washing of potentially contaminated clothing has been included in the plan (40°C for heat sensitive and 60°C for heat resistant).		☐	☐	☐
A procedure has been developed for personnel to remove their work clothes after the workday.		☐	☐	☐
▪ Describe the pathway and procedures				
Cleaning plan includes a process for waste disposal, including personal protective equipment (PPE) and diapers (biohazard waste).		☐	☐	☐
▪ Describe the process of waste disposal				
Alcohol-based hand sanitizer for hand hygiene is available in every resident room (inside and out), other common areas and hallways.		☐	☐	☐
Sinks are well-stocked with soap and paper towels for hand washing.		☐	☐	☐
Signs about procedures to prevent the dissemination of infection are available throughout the facility (e.g. respiratory etiquette, hand washing, air renewal every 6 hours).		☐	☐	☐
The facility provides tissues and facemasks for coughing people (residents and personnel) in common areas with no-touch receptacles for disposal.		☐	☐	☐
Necessary personal protective equipment (PPE) is available outside of the resident room and in other areas where resident care is provided.		☐	☐	☐
PPE availability	Gowns	☐	Surgical mask	☐
	Glasses/Face shields	☐	Cloth masks	☐
List others, if any:	Gloves	☐	FPP2/FPP3 masks	☐
	Cap	☐	Cover boots	☐
The facility has a process to monitor supply levels (e.g. PPE and disinfectant products).		☐	☐	☐

4. Elements of a COVID-19 plan		Completed	In progress	Not initiated
The facility has ensured in the contingency plan processes that could be triggered when they experience supply shortages (PPE and disinfectant products).		☐	☐	☐
Notes				
**4.5. Education and training**				
A person has been designated with responsibility for coordinating education and training on COVID-19.		☐	☐	☐
An education and training plan has been developed to prevent new cases of coronavirus infection; the target population of this education and training plan are:				
▪ the facility's personnel		☐	☐	☐
▪ the facility's external collaborators		☐	☐	☐
▪ the new staff hired to bolster the facility's personnel		☐	☐	☐
▪ the volunteers		☐	☐	☐
▪ the health professionals at the facility		☐	☐	☐
▪ the residents		☐	☐	☐
▪ the family members and visitors		☐	☐	☐
Any training and awareness materials have been developed taking into consideration the characteristics of the target audience (e.g., reading-level, health literacy).		☐	☐	☐
The facility keeps a record of all education and training activities related with the adequate use of personal protective equipment (PPE).		☐	☐	☐
Notes				
**4.6. Occupational health**				
The facility has sick leave policies that are non-punitive and consistent with public health policies that allow personnel to stay home in case of need.		☐	☐	☐
Notes				
The facility has developed a plan for the distribution and rotation of work schedules among personnel (e.g. 14-day at work rotation).		☐	☐	☐
Notes				
The facility instructs the personnel to regularly monitor themselves for any symptoms related with coronavirus infection		☐	☐	☐
Notes				
The facility has a process to actively screen residents and their family members/visitors for any symptoms related with coronavirus infection.		☐	☐	☐
Notes				

4. Elements of a COVID-19 plan	Completed	In progress	Not initiated
The facility has a process for actively screen personnel for symptoms of a coronavirus infection when they report to work.	☐	☐	☐
Notes			
The facility has a process for actively screen personnel for symptoms of a coronavirus infection at the end of the workday.	☐	☐	☐
Notes			
The personnel was involved in decision-making on matters that directly or indirectly affect their routine practice, and the regular work processes and procedures.	☐	☐	☐
Notes			
The facility has a process to monitor the implementation and the effects of measures implemented in the facility to prevent potential contagion situations.	☐	☐	☐
Notes			
The measures in force in the facility imply:			
▪ that common spaces are only used by residents and personnel without symptoms of (acute) respiratory infection.	☐	☐	☐
▪ a physical distance of at least 1.5 meters between people.	☐	☐	☐
▪ the use of common spaces to be done in shifts, including meal periods.	☐	☐	☐
▪ the provision of beds spaced at least 1.5 meters apart.	☐	☐	☐
▪ that suspected COVID-19 cases do not share the same space of confirmed cases.	☐	☐	☐
▪ that suspected or confirmed COVID-19 cases do not share common spaces with fellow residents and personnel.	☐	☐	☐
▪ the possibility of transferring residents to other facilities (e.g. hotel).	☐	☐	☐
▪ restriction or cancellation of group activities.	☐	☐	☐
▪ the provision of means of contact so that residents can communicate with family members.	☐	☐	☐
▪ List others, if any:	☐	☐	☐
Notes			
**4.7. Identification and management of ill residents**			
The facility has developed a plan for isolating suspected or confirmed cases of coronavirus infection.	☐	☐	☐
The plan includes the processes and procedures on how to immediately notify the competent authorities on suspected cases of coronavirus infection.	☐	☐	☐
The admission process for new residents has been revised and requires:			

4. Elements of a COVID-19 plan	Completed	In progress	Not initiated
▪ a negative laboratory test for SARS-CoV-2.	☐	☐	☐
▪ that all new residents show no signs and symptoms of respiratory infection.	☐	☐	☐
▪ a mandatory isolation period of 14 days (minimum).	☐	☐	☐
The facility has developed a plan for when a resident leaves the institution for less than 24 hours (e.g. isolation period of at least 14 days).	☐	☐	☐
The facility has developed a plan for when a resident leaves the institution for more than 24 hours (e.g. laboratory test for SARS-CoV-2).	☐	☐	☐
Notes			
**4.8. Access control**			
The institution has developed or used materials at all entrances to signal access restriction to the facility to all individuals with febrile symptoms or with symptoms of respiratory infection.	☐	☐	☐
The facility has developed a plan for non-essential visits and where potential restrictions apply, those affected were informed	☐	☐	☐
The facility has developed a plan to handle supplier that:			
▪ require access to the interior of the facility.	☐	☐	☐
▪ do not require access to the interior of the facility, and thus, a (un)loading area was defined outside the facility.	☐	☐	☐
Notes			

5. Are there items that you would like to have implemented in the institution, but that due to the difficulty in gathering reliable information, access to adequate training, or availability of resources was not possible? If so, specify.

6. Other comments

Thank you for collaborating. We are at your service.
