# Supplementary material for: COVID-19 Preparedness and Perceived Safety in Nursing Homes in Southern Portugal: A Cross-Sectional Survey-Based Study in the Initial Phases of the Pandemic
Source: Int J Environ Res Public Health. 2021 Jul 28;18(15):7983. doi: 10.3390/ijerph18157983 (PMC8345424; doi:10.3390/ijerph18157983)
Supplement: Supplementary file 1 [file ijerph-18-07983-s001.zip › File S2.pdf]

Supplementary File 2

<table>
<tr><th colspan="6">Nursing home COVID-19 preparedness checklist
Follow-up conversation note taking template</th></tr>
<tr><th colspan="6">1. Institution</th></tr>
<tr><td>Name</td><td></td><td>COVID 70+ ID</td><td></td><td>Start (h:m)</td><td></td></tr>
<tr><td>Institution’s representative</td><td></td><td rowspan="2">Date</td><td rowspan="2"></td><td rowspan="2">End (h:m)</td><td rowspan="2"></td></tr>
<tr><td>COVID 70+ representative</td><td></td></tr>
</table>

<table>
<tr><th colspan="5">NOTES
(The notes should focus on the aspects of each of the areas covered in checklist and provide additional information on the difficulties in implementing measures, diagnosis of current needs and potential solutions)</th></tr>
<tr><th colspan="5">1. Personnel structure</th></tr>
<tr><td colspan="2">Management
(e.g. unit directors, coordinators)</td><td></td><td>Medical staff</td><td></td></tr>
<tr><td colspan="2">Administrative support
(e.g. human resources, secretariat)</td><td></td><td>Nursing staff</td><td></td></tr>
<tr><td colspan="2">General support
(e.g. drivers, maintenance, security)</td><td></td><td>Health care assistants</td><td></td></tr>
<tr><td>Others</td><td></td><td></td><td>Other care providers
(e.g. nutritionist, therapists, social support)</td><td></td></tr>
<tr><td colspan="2">General remarks</td><td colspan="3"></td></tr>
<tr><th colspan="5">2. Structure for planning and decision making</th></tr>
<tr><td colspan="5"></td></tr>
<tr><th colspan="5">3. COVID-19 contingency plan</th></tr>
<tr><td colspan="5"></td></tr>
<tr><th colspan="5">4. Elements of a COVID-19 plan</th></tr>
<tr><td colspan="2">4.1. General</td><td colspan="3"></td></tr>
<tr><td colspan="2">4.2. Outbreak capacity</td><td colspan="3"></td></tr>
<tr><td colspan="2">4.3. Communication</td><td colspan="3"></td></tr>
<tr><td colspan="2">4.4. Supplies and resources</td><td colspan="3"></td></tr>
<tr><td colspan="2">4.5. Education and training</td><td colspan="3"></td></tr>
<tr><td colspan="2">4.6. Occupational health</td><td colspan="3"></td></tr>
<tr><td colspan="2">4.7. Identification and management of ill residents</td><td colspan="3"></td></tr>
<tr><td colspan="2">4.8. Access control</td><td colspan="3"></td></tr>
</table>

5. Identification of rapid implementation measures and respective prioritization given the characteristics of the facility.

6. Further comments from the COVID 70+ project representative
