# Supplementary material for: COVID-19 Preparedness and Perceived Safety in Nursing Homes in Southern Portugal: A Cross-Sectional Survey-Based Study in the Initial Phases of the Pandemic
Source: Int J Environ Res Public Health. 2021 Jul 28;18(15):7983. doi: 10.3390/ijerph18157983 (PMC8345424; doi:10.3390/ijerph18157983)
Supplement: Supplementary file 1 [file ijerph-18-07983-s001.zip › File S3.pdf]

Supplementary File 3

SAFETY CONCERNS AND WELL-BEING OF NURSING HOME PERSONNEL

EN Version: May 10, 2020
Target: Nursing home personnel
Mode: Self-administered web-based survey
Original in the Portuguese language

SECTION

Recently, a team of doctors and nurses of the COVID 70+ project, a partnership between the Algarve Biomedical Center, University of the Algarve and the Ministry of Labor, Solidarity and Social Security, have been at your nursing home to test the residents and the personnel for the virus that causes COVID-19. This survey is being conducted by team members of the COVID 70+ project **to better understand safety concerns and well-being of nursing home professionals in the context of the coronavirus pandemic.**

We kindly invite you, as a nursing home worker, to voluntarily answer this **anonymous** survey, which is expected to take approximately **15 minutes to complete**. By having your responses to this survey, we intend to support **nursing homes and other key organizations to strengthen their responses to crisis situations.** Your collaboration is, therefore, very important.

There are no known risks to you for participating in this survey. You will not be required to provide identifiable information. The results of the survey may be featured in project reports or scientific articles, but **you will never be identified**.

If you have any questions about this survey or the COVID 70+ project, you may contact at any time the coordination team: coordenacao.drive2.homecare@abcmedicalg.pt

Many thanks for your participation!

SECTION

Q1
Do you accept to take part in this survey and confirm that you are aged 18 and older, aware of the terms of your participation and that you are well informed on your rights?
( ) YES
( ) NO → End of survey

SECTION

The following questions ask how you perceive your risk of becoming affected with the virus that causes COVID-19, given your circumstances (e.g. pre-existing health-related conditions, your profession or the adoption of recommended behaviors by health authorities), on a scale from 0 to 10. Zero means you perceive the risk of becoming infected is "very low", and 10 means "very high".

Q2
How do you perceive your risk of becoming infected with the virus that causes COVID-19?

Very low										Very high
0	1	2	3	4	5	6	7	8	9	10

Q3
Compared to others, how do you perceive your risk of becoming severely ill if you would become infected with the virus that causes COVID-19?

Very low										Very high
0	1	2	3	4	5	6	7	8	9	10

Q4
Please indicate how often did the following things happen with other Staff working with you at your nursing home during the LAST MONTH.

	Over the last month	All of the time	Most of the time	More than half of the time	Less than half of the time	Some of the time	At no time	I don't know/prefer not to answer
1	**Staff have felt fear of becoming infected with the virus that causes COVID-19 because of their job-related tasks**	☐	☐	☐	☐	☐	☐	☐
2	**Staff have felt fear of infecting nursing home**	☐	☐	☐	☐	☐	☐	☐

	**residents or colleagues while at work**							
3	**Staff have felt fear of infecting their family members**	☐	☐	☐	☐	☐	☐	☐
4	**Staff have felt confident with their training in using personal protective equipment**	☐	☐	☐	☐	☐	☐	☐
5	**Staff have been filled with anxiety because of the use of personal protective equipment at work**	☐	☐	☐	☐	☐	☐	☐
6	**Staff were trustful that the stocks of personal protective equipment at work would be enough**	☐	☐	☐	☐	☐	☐	☐
7	**Staff have been missing work out of fear of becoming infected with the virus that causes COVID-19**	☐	☐	☐	☐	☐	☐	☐
8	**Staff have been missing work out of fear of infecting people at their household**	☐	☐	☐	☐	☐	☐	☐
9	**Staff have been missing work because they were recommended to stay at home in quarantine**	☐	☐	☐	☐	☐	☐	☐
10	**Staff have been missing work to provide assistance to family members (e.g. taking care of children)**	☐	☐	☐	☐	☐	☐	☐

SECTION

The following question asks how important it was to you and the residents at your nursing home to be tested for COVID-19, under the COVID70+ project, on a scale from 0 to 10. Zero means you feel that it was “not at all important” and 10 means you feel that it was “very important”.

Q5
Were you tested for COVID-19 at the nursing home where you work at?
(   ) YES
(   ) NO → Jump to Q7

Q6
How important was it to you to be tested for COVID-19?

Not at all important										Very important
0	1	2	3	4	5	6	7	8	9	10

Q7
How important was it to you to have the residents at your nursing home tested for COVID-19?

Not at all important										Very important
0	1	2	3	4	5	6	7	8	9	10

Q8
Have you been dealing with individuals infected with the virus that causes COVID-19 that you are aware of?
(   ) YES → Jump to Q9
(   ) NO → Jump to Q11
(   ) I DON'T KNOW → Jump to Q10
(   ) PREFER NOT TO ANSWER → Jump to Q11

Q9
Where have you been dealing with these individuals? Mark all that apply.
(   ) AT THIS NURSING HOME → Jump to Q11
(   ) AT ANOTHER WORKPLACE → Jump to Q11
(   ) AT MY HOME → Jump to Q11
(   ) AT THE HOME OF A RELATIVE → Jump to Q11
(   ) ANOTHER PLACE → Jump to Q11
(   ) PREFER NOT TO ANSWER → Jump to Q11

Q10
Where can you have dealt with these individuals? Mark all that apply.
(   ) AT THIS NURSING HOME → Jump to Q11
(   ) AT ANOTHER WORKPLACE → Jump to Q11
(   ) AT MY HOME → Jump to Q11
(   ) AT THE HOME OF A RELATIVE → Jump to Q11
(   ) ANOTHER PLACE → Jump to Q11
(   ) PREFER NOT TO ANSWER → Jump to Q11

SECTION
[Part of the AHRQ's Nursing home survey on patient safety culture]

Q11
How much do you agree or disagree with the following statements?

If a question does not apply to your current job or you do not know the answer, please mark the box in the last column (on your right side).

How much do you agree or disagree with the following statements?	Strongly Disagree ▼	Disagree ▼	Neither Agree nor Disagree ▼	Agree ▼	Strongly Agree ▼	Does Not Apply or Don't Know ▼
1. Staff in this nursing home treat each other with respect..............................	☐1	☐2	☐3	☐4	☐5	☐9
2. Staff support one another in this nursing home....................................	☐1	☐2	☐3	☐4	☐5	☐9
3. We have enough staff to handle the workload	☐1	☐2	☐3	☐4	☐5	☐9
4. Staff follow standard procedures to care for residents...............................	☐1	☐2	☐3	☐4	☐5	☐9
5. Staff feel like they are part of a team.	☐1	☐2	☐3	☐4	☐5	☐9
6. Staff use shortcuts to get their work done faster..	☐1	☐2	☐3	☐4	☐5	☐9
7. Staff get the training they need in this nursing home..............................	☐1	☐2	☐3	☐4	☐5	☐9
8. Staff have to hurry because they have too much work to do	☐1	☐2	☐3	☐4	☐5	☐9
9. When someone gets really busy in this nursing home, other staff help out	☐1	☐2	☐3	☐4	☐5	☐9
10. Staff are blamed when a resident is harmed	☐1	☐2	☐3	☐4	☐5	☐9
11. Staff have enough training on how to handle difficult residents....................	☐1	☐2	☐3	☐4	☐5	☐9
12. Staff are afraid to report their mistakes	☐1	☐2	☐3	☐4	☐5	☐9
13. Staff understand the training they get in this nursing home	☐1	☐2	☐3	☐4	☐5	☐9

14. To make work easier, staff often ignore procedures	☐1	☐2	☐3	☐4	☐5	☐9
15. Staff are treated fairly when they make mistakes	☐1	☐2	☐3	☐4	☐5	☐9
16. Residents' needs are met during shift changes...	☐1	☐2	☐3	☐4	☐5	☐9
17. It is hard to keep residents safe here because so many staff quit their jobs	☐1	☐2	☐3	☐4	☐5	☐9
18. Staff feel safe reporting their mistakes	☐1	☐2	☐3	☐4	☐5	☐9

SECTION
[WHO well-being 5-item index]

The following question asks how satisfied you feel, on a scale from 0 to 10. Zero means you feel "not at all satisfied" and 10 means you feel "completely satisfied".

Q12
Overall, how satisfied are you with life as a whole these days?

Not at all satisfied										Completely satisfied
0	1	2	3	4	5	6	7	8	9	10

Q13
Please indicate for each of the five statements which is closest to how you have been feeling over the last two weeks.
Notice that higher numbers mean better well-being.

	Over the last two weeks	All of the time	Most of the time	More than half of the time	Less than half of the time	Some of the time	At no time
1	**I have felt cheerful and in good spirits**	☐ 5	☐ 4	☐ 3	☐ 2	☐ 1	☐ 0
2	**I have felt calm and relaxed**	☐ 5	☐ 4	☐ 3	☐ 2	☐ 1	☐ 0
3	**I have felt active and vigorous**	☐ 5	☐ 4	☐ 3	☐ 2	☐ 1	☐ 0
4	**I woke up feeling fresh and rested**	☐ 5	☐ 4	☐ 3	☐ 2	☐ 1	☐ 0
5	**My daily life has been filled with things that interest me**	☐ 5	☐ 4	☐ 3	☐ 2	☐ 1	☐ 0

SECTION

Q14

What is your job in this nursing home?

Check ONE box that best applies to your job. If more than one category applies, check the highest-level job.

(   ) Administrator/Manager

(   ) Social worker

(   ) Occupational worker

(   ) Physical therapist

(   ) Physician

(   ) Nurse

(   ) Nutritionist/dietitian

(   ) Speech therapist

(   ) Sociocultural worker

(   ) Administrative

(   ) Receptionist

(   ) Driver

(   ) Cleaning and maintenance

(   ) Other → Jump to Q15

Q15
Please write the title of your job. ____________

Q16
How long have you worked in this nursing home?

( ) Less than 2 months

( ) 2 to 11 months

( ) 1 to 2 years

( ) 3 to 5 years

( ) 6 to 10 years

( ) 11 years or more

Q17
In this nursing home, do you work directly with residents most of the time?
( ) YES
( ) NO

Q18
How many hours per week do you usually work in this nursing home?
( ) 10 or less hours per week

( ) 11 to 20 hours per week

( ) 21 to 30 hours per week

( ) 31 to 40 hours per week

( ) More than 40 hours per week

SECTION
[Includes the Minimum European Health Module]

Q19
How is your health in general?
( ) VERY GOOD
( ) GOOD
( ) FAIR
( ) BAD
( ) VERY BAD

Q20
Do you have any longstanding illness or health problem?
( ) YES
( ) NO
( ) NOT SURE
( ) PREFER NOT TO ANSWER

Q21

For at least the past 6 months, to what extent have you been limited because of a health problem in activities people usually do?
( ) SEVERELY LIMITED
( ) LIMITED BUT NOT SEVERELY
( ) NOT LIMITED AT ALL
( ) NOT SURE
( ) PREFER NOT TO ANSWER

Q22
Age: _____

Q23
What is your sex?
( ) FEMALE
( ) MALE
( ) PREFER NOT TO ANSWER

Q24
What is your highest level of education?
( ) LESS THAN THE 4th GRADE
( ) 4th GRADE
( ) 6th GRADE
( ) 9th GRADE
( ) 12th GRADE
( ) HIGHER EDUCATION
( ) PREFER NOT TO ANSWER

Q25
Do you live with an older person (65 years old and over) or an individual with any longstanding illness or health problem?
( ) YES
( ) NO
( ) PREFER NOT TO ANSWER

Q26
Do you usually take care of any children or grandchildren (younger than 12 years old) who may be under your responsibility to be taken care of?
( ) YES
( ) NO
( ) PREFER NOT TO ANSWER

Q27

Do you live with someone that belongs to a professional group that is more at risk of being infected with the virus that causes COVID-19 (e.g. health care professional or other that could be considered an essential worker)?

( ) YES
( ) NO
( ) PREFER NOT TO ANSWER

Q28
In which area do you feel a need for support from others? Mark all that apply.

( ) I DO NOT FEEL A NEED FOR SUPPORT FROM OTHERS
( ) DEALING WITH MY OWN NEGATIVE MOOD
( ) DEALING WITH STRESS
( ) DEALING WITH FEAR/ANXIETY
( ) DEALING WITH THE EMOTIONS OF OTHERS
( ) TO OCCUPY MYSELF WITH OTHER ACTIVITES
( ) SLEEPING
( ) DEALING WITH GRIEF
( ) OTHER → Jump to Q29

Q29
If you feel a need for support from other in other areas, please indicate which.

SECTION

Q30
If there is anything else you would like to tell us about your experience during this pandemic crisis, please do so here. Your comments will be looked at in full, but we will discard any information that could identify you.

SECTION

Q31
Do you wish to be reported on a summary of the results of this survey?

( ) YES → Jump to Q32
( ) NO → End of survey

Q32

In which e-mail address you prefer to receive that information?

SECTION

END OF SURVEY
