# Supplementary material for: COVID-19 Preparedness and Perceived Safety in Nursing Homes in Southern Portugal: A Cross-Sectional Survey-Based Study in the Initial Phases of the Pandemic
Source: Int J Environ Res Public Health. 2021 Jul 28;18(15):7983. doi: 10.3390/ijerph18157983 (PMC8345424; doi:10.3390/ijerph18157983)
Supplement: Supplementary file 1 [file ijerph-18-07983-s001.zip › File S4.pdf]

Supplementary File 4

Nursing home COVID-19 preparedness checklist compliance scores (breakdowns)

Item	Description	Algarve (n=51)		Alentejo (n=87)		Total (n=138)	
		n	%	n	%	n	%
**2 | Structure for planning and decision making**							
2.1.	Response to COVID-19 has been incorporated into emergency management planning for the facility	29	57%	64	74%	93	67%
2.2.	A multidisciplinary planning team has been created to specifically address COVID-19 preparedness planning and monitoring	31	61%	63	72%	94	68%
2.3.	A COVID-19 response coordinator has been assigned	41	80%	80	92%	121	88%
**3 | COVID-19 contingency plan**							
3.1.	There is a contingency plan in place that introduces mechanisms to protect the health of users, professionals and potential visitors	43	84%	78	90%	121	88%
3.2.	A copy of the COVID-19 contingency plan is easily available at the facility and accessible by personnel	36	71%	82	94%	118	86%
3.3.	A COVID-19 information monitoring mechanism is available for continuously update of the contingency plan (for example, guidelines from the Directorate-General of Health)	40	78%	74	85%	114	83%
3.4.	The plan identifies the person(s) authorized to implement the plan and the management, monitoring and evaluation structure that will be used	35	69%	69	79%	104	75%
**4 | Elements of a COVID-19 plan**							
**4.1. General**							
4.1.1.	A person has been assigned responsibility for monitoring information set forth by the competent public health authorities and updating the COVID-19 response coordinator in the facility	46	90%	77	89%	123	89%
4.1.2.	The facility has a process for inter-facility transfers that includes notifying personnel and receiving facilities about a resident's suspected or confirmed diagnosis	28	55%	56	64%	84	61%
4.1.3.	The facility has a system for monitoring the evolution of COVID-19 among residents and personnel in the facility. Information from this monitoring system is used to implement prevention interventions	39	76%	56	64%	95	69%

Item	Description	Algarve (n=51)		Alentejo (n=87)		Total (n=138)	
		n	%	n	%	n	%
4.1.4.	The facility has infection control policies that outline the precautions recommended to residents and personnel, including the dissemination of information and use of specific signs (e.g., posters and leaflets)	44	86%	79	91%	123	89%
4.1.5.	A mechanism for updating the COVID-19 response plan is well defined, including any deadlines	20	39%	49	56%	69	50%
**4.2. Outbreak capacity**							
4.2.1.	A contingency personnel plan has been developed that identifies the minimum personnel needs and prioritizes essential services based on residents' characteristics (e.g., functional limitations)	32	63%	65	75%	97	70%
4.2.2.	A person has been assigned responsibility for a daily assessment and monitoring of personnel status and needs during a COVID-19 outbreak	39	76%	72	83%	111	80%
4.2.3.	Estimates have been made of the quantities of materials and equipment that would be needed during a minimum four-week outbreak	32	63%	66	76%	98	71%
4.2.4.	A rationing strategy has been developed for Personal Protective Equipment (PPE), medicines and other scarce materials	34	67%	70	80%	104	75%
4.2.5.	A contingency plan has been developed for managing an increased need for postmortem care and disposition of deceased residents	9	18%	26	30%	35	25%
4.2.6.	An area in the facility that could be used as a temporary morgue has been identified	11	22%	27	31%	38	28%
4.2.7.	Any plan for expanding the morgue capacity plan been discussed and decided with the competent authorities	4	8%	9	10%	13	9%
**4.3. Communication**							
4.3.1.	Key public health points of contact during the stages of the COVID-19 pandemic have been identified	44	86%	75	86%	119	86%
4.3.2.	Communication plans include how visitors should be informed about the importance of monitoring symptoms for 14 days after visiting the facility	37	73%	47	54%	84	61%
4.3.3.	A person has been assigned responsibility for communications with personnel, residents and their families regarding the status, preparedness and impact of COVID-19 in the facility	45	88%	80	92%	125	91%
4.3.4.	Contact information and communication channels for family members of facility residents are up to date and fully disseminated	47	92%	78	90%	125	91%

Item	Description	Algarve (n=51)		Alentejo (n=87)		Total (n=138)	
		n	%	n	%	n	%
4.3.5.	Communication plans include how signs and other methods of communication will be used across the facility	30	59%	57	66%	87	63%
**4.4. Supplies and resources**							
4.4.1.	A written and detailed plan for regular cleaning and disinfection of the facility has been developed	33	65%	61	70%	94	68%
4.4.2.	The cleaning and disinfection of the facility is ensured by a specialized company whenever confirmed cases are reported	8	16%	32	37%	40	29%
4.4.3	The cleaning and disinfection products used at the facility adhere to recommended standards	47	92%	82	94%	129	93%
4.4.4.	All cleaning and disinfection products in use at the facility are well described in the cleaning and disinfection plan, and they are properly labeled, packaged and stored	37	73%	65	75%	102	74%
4.4.5.	The facility has access to disinfectant that is suitable for hospital use for cleaning the surfaces and equipment	37	73%	78	90%	115	83%
4.4.6.	A regular cleaning and disinfection plan for bedpans, urinals and toilet bowls for residents with suspected or confirmed infection has been included in the plan	36	71%	65	75%	101	73%
4.4.7	The cleaning and disinfection of the most frequently used surfaces (e.g., tables and chairs) has been included in the plan	43	84%	76	87%	119	86%
4.4.8.	The cleaning and disinfection of shared equipment (e.g., thermometers) has been included in the plan	46	90%	77	89%	123	89%
4.4.9.	The cleaning and washing of potentially contaminated clothing have been included in the plan (40°C for heat sensitive and 60°C for heat resistant)	45	88%	71	82%	116	84%
4.4.10	A procedure has been developed for personnel to remove their work clothes after the workday	40	78%	70	80%	110	80%
4.4.11	Cleaning plan includes a process for waste disposal, including personal protective equipment (PPE) and diapers (biohazard waste)	28	55%	56	64%	84	61%
4.4.12	Alcohol-based hand sanitizer for hand hygiene is available in every resident room (inside and out), other common areas and hallways	46	90%	81	93%	127	92%
4.4.13	Sinks are well-stocked with soap and paper towels for hand washing	49	96%	85	98%	134	97%
4.4.14	Signs about procedures to prevent the dissemination of infection are available throughout the facility (e.g. respiratory etiquette, hand washing, air renewal every 6 hours)	43	84%	84	97%	127	92%

Item	Description	Algarve (n=51)		Alentejo (n=87)		Total (n=138)	
		n	%	n	%	n	%
4.4.15	The facility provides tissues and facemasks for coughing people (residents and personnel) in common areas with no-touch receptacles for disposal	44	86%	83	95%	127	92%
4.4.16	Necessary personal protective equipment (PPE) is available outside of the resident room and in other areas where resident care is provided	37	73%	81	93%	118	86%
4.4.17	The facility has a process to monitor supply levels (e.g. PPE and disinfectant products)	36	71%	70	80%	106	77%
4.4.18	The facility has ensured in the contingency plan processes that could be triggered when they experience supply shortages (PPE and disinfectant products)	16	31%	45	52%	61	44%
**4.5. Education and training**							
4.5.1	A person has been designated with responsibility for coordinating education and training on COVID-19	32	63%	57	66%	89	64%
4.5.2.1	An education and training plan has been developed to prevent new cases of coronavirus infection; the target population of this education and training plan are the facility's personnel	32	63%	59	68%	91	66%
4.5.2.2	An education and training plan has been developed to prevent new cases of coronavirus infection; the target population of this education and training plan are the facility's external collaborators	16	31%	27	31%	43	31%
4.5.2.3	An education and training plan has been developed to prevent new cases of coronavirus infection; the target population of this education and training plan are the new staff hired to bolster the facility's personnel	24	47%	30	34%	54	39%
4.5.2.4	An education and training plan has been developed to prevent new cases of coronavirus infection; the target population of this education and training plan are volunteers	16	31%	20	23%	36	26%
4.5.2.5	An education and training plan has been developed to prevent new cases of coronavirus infection; the target population of this education and training plan are the health professionals at the facility	31	61%	57	66%	88	64%
4.5.2.6	An education and training plan has been developed to prevent new cases of coronavirus infection; the target population of this education and training plan are the residents	30	59%	44	51%	74	54%
4.5.2.7	An education and training plan has been developed to prevent new cases of coronavirus infection; the target population of this education and training plan are the family members and visitors	15	29%	27	31%	42	30%

Item	Description	Algarve (n=51)		Alentejo (n=87)		Total (n=138)	
		n	%	n	%	n	%
4.5.3	Any training and awareness materials have been developed taking into consideration the characteristics of the target audience (e.g., reading-level, health literacy)	17	33%	37	43%	54	39%
4.5.4	The facility keeps a record of all education and training activities related with the adequate use of personal protective equipment (PPE)	21	41%	37	43%	58	42%
**4.6. Occupational health**							
4.6.1	The facility has sick leave policies that are non-punitive and consistent with public health policies that allow personnel to stay home in case of need	39	76%	67	77%	106	77%
4.6.2	The facility has developed a plan for the distribution and rotation of work schedules among personnel (e.g., 14-day at work rotation)	27	53%	65	75%	92	67%
4.6.3	The facility instructs the personnel to regularly monitor themselves for any symptoms related with coronavirus infection	46	90%	77	89%	123	89%
4.6.4	The facility has a process to actively screen residents and their family members/visitors for any symptoms related with coronavirus infection	47	92%	73	84%	120	87%
4.6.5	The facility has a process for actively screen personnel for symptoms of a coronavirus infection when they report to work	42	82%	64	74%	106	77%
4.6.6	The facility has a process for actively screen personnel for symptoms of a coronavirus infection at the end of the workday	35	69%	55	63%	90	65%
4.6.7	The personnel was involved in decision-making on matters that directly or indirectly affect their routine practice, and the regular work processes and procedures	44	86%	78	90%	122	88%
4.6.8	The facility has a process to monitor the implementation and the effects of measures implemented in the facility to prevent potential contagion situations	30	59%	60	69%	90	65%
4.6.9.1	The measures in force in the facility imply that common spaces are only used by residents and personnel without symptoms of (acute) respiratory infection	43	84%	72	83%	115	83%
4.6.9.2	The measures in force in the facility imply a physical distance of at least 1.5 meters between people	37	73%	63	72%	100	72%
4.6.9.3	The measures in force in the facility imply the use of common spaces to be done in shifts, including meal periods.	34	67%	54	62%	88	64%

Item	Description	Algarve (n=51)		Alentejo (n=87)		Total (n=138)	
		n	%	n	%	n	%
4.6.9.4	The measures in force in the facility imply the provision of beds spaced at least 1.5 meters apart	34	67%	56	64%	90	65%
4.6.9.5	The measures in force in the facility imply that suspected COVID-19 cases do not share the same space of confirmed cases	41	80%	75	86%	116	84%
4.6.9.6	The measures in force in the facility imply that suspected or confirmed COVID-19 cases do not share common spaces with fellow residents and personnel	38	75%	76	87%	114	83%
4.6.9.7	The measures in force in the facility foresee the possibility of transferring residents to other facilities (e.g., hotel)	12	24%	30	34%	42	30%
4.6.9.8	The measures in force in the facility imply restriction or cancellation of group activities	48	94%	81	93%	129	93%
4.6.9.9	The measures in force in the facility imply the provision of means of contact so that residents can communicate with family members	48	94%	84	97%	132	96%
**4.7. Identification and management of ill residents**							
4.7.1	The facility has developed a plan for isolating suspected or confirmed cases of coronavirus infection	46	90%	82	94%	128	93%
4.7.2	The plan includes the processes and procedures on how to immediately notify the competent authorities on suspected cases of coronavirus infection	47	92%	81	93%	128	93%
4.7.3.1	The admission process for new residents has been revised and requires a negative laboratory test for SARS-CoV-2	45	88%	69	79%	114	83%
4.7.3.2	The admission process for new residents has been revised and requires that all new residents show no signs and symptoms of respiratory infection	44	86%	65	75%	109	79%
4.7.3.3	The admission process for new residents has been revised and requires a mandatory isolation period of 14 days (minimum)	45	88%	71	82%	116	84%
4.7.4	The facility has developed a plan for when a resident leaves the institution for less than 24 hours (e.g., isolation period of at least 14 days)	44	86%	63	72%	107	78%
4.7.5	The facility has developed a plan for when a resident leaves the institution for more than 24 hours (e.g., laboratory test for SARS-CoV-2)	41	80%	63	72%	104	75%
**4.8. Access control**							
4.8.1	The institution has developed or used materials at all entrances to signal access restriction to the facility to all individuals with febrile symptoms or with symptoms of respiratory infection	42	82%	60	69%	102	74%

Item	Description	Algarve (n=51)		Alentejo (n=87)		Total (n=138)	
		n	%	n	%	n	%
4.8.2	The facility has developed a plan for non-essential visits and where potential restrictions apply, those affected were informed	42	82%	79	91%	121	88%
4.8.3.1	The facility has developed a plan to handle supplier that require access to the interior of the facility	41	80%	68	78%	109	79%
4.8.3.2	The facility has developed a plan to handle suppliers that do not require access to the interior of the facility, and thus, a (un)loading area was defined outside the facility	45	88%	78	90%	123	89%
