# Supplementary material for: COVID-19 Preparedness and Perceived Safety in Nursing Homes in Southern Portugal: A Cross-Sectional Survey-Based Study in the Initial Phases of the Pandemic
Source: Int J Environ Res Public Health. 2021 Jul 28;18(15):7983. doi: 10.3390/ijerph18157983 (PMC8345424; doi:10.3390/ijerph18157983)
Supplement: Supplementary file 1 [file ijerph-18-07983-s001.zip › File S5.pdf]

Supplementary File 5

Key underdeveloped COVID-19 preparedness checklist items and good practices among nursing homes

COVID-19 preparedness categories	Common underdeveloped items	Identification of good practices
**Structure for planning and decision making**	▪ Insufficient multidisciplinary governance structure.<br>▪ Governance structure not clearly defined.	▪ Governance structure for planning and decision-making includes the nursing home director, clinical director, technical director, health care professional (nurses and a psychologist) and a social worker.<br>▪ Maintenance of an up-to-date emergency contact list, listing relevant regional and local health and other competent authorities' contacts.
**Development of a written COVID-19 contingency plan**	▪ Underdeveloped contingency plan (e.g., insufficient planning on how to deal in case of a confirmed or suspicious case).<br>▪ Unawareness of the existence of a contingency plan and its details by key stakeholders (e.g., nursing home personnel).<br>▪ No periodic revision of the contingency plan.	▪ The contingency plan is continuously revised based on the guidelines of the Directorate-General of Health and other relevant competent authorities.<br>▪ Access-restricted WhatsApp channel to share and discuss aspects of the contingency plan (e.g., updates based on novel Directorate-General of Health guidelines).
**General aspects of a COVID-19 plan**	▪ Poor planning to isolate and/or transfer residents, if needed.<br>▪ Poor surveillance mechanisms to monitor symptoms among residents and staff that could signal an eventual coronavirus infection.	▪ Clear identification of a key person responsible for monitoring guideline updates and relaying it to the governance team.<br>▪ Designation of a shift team leader responsible of activating emergency mechanisms foreseen in the contingency plan.

COVID-19 preparedness categories	Common underdeveloped items	Identification of good practices
**Outbreak capacity**	▪ Insufficient planning addressing infrastructure constraints (e.g., insufficient space for isolation rooms).<br>▪ Insufficient planning to overcome absenteeism hurdles.<br>▪ Insufficient planning to overcome financial and market availability constraints to personal protective equipment (PPE).	▪ Having an emergency protocol with the nearest primary health care centers to be activated in the case of an outbreak.
**Facility communications**	▪ Inadequate communication with families and carers, including the monitoring of symptoms when these people still had access to the facilities.<br>▪ Flawed communication channels with health and other competent authorities.	▪ The use of social media and other platforms for updating families and carers on the nursing home's planning and initiatives to mitigate the effects of the pandemic.
**Supplies and resources**	▪ Insufficient detail of the cleaning plan and inexistence of written records to keep track of cleaned areas.<br>▪ Generalized shortage of specific PPE (e.g., gowns and FFP2 masks)<br>▪ Improper use and handling of PPE.	▪ Contracting of a professional cleaning services with biohazard waste collection in case of positive cases of COVID-19.<br>▪ Systematic inventory and maintenance of sufficient stock of PPE, in close collaboration with governmental authorities.
**Education and training**	▪ No specific education and training planning, with clear focus and objectives, and towards the needs of different audiences	▪ Identification and training of experienced volunteers who could be called upon a shortage of personnel.<br>▪ Training programes conceptualized and implemented by experienced trainers with adequate certification.

COVID-19 preparedness categories	Common underdeveloped items	Identification of good practices
**Occupational health**	▪ Inexistence of a monitoring system to assess the implementation and effects of measures at the institutional and individual level (residents and personnel), such as the implementation of 14-day rotating shifts among personnel and the cancellation of all visiting hours.<br>▪ Clean/unclean circuits lack detail and are not well signaled throughout the facility.<br>▪ Nonpunitive culture regarding leaves off of work are overlooked in the contingency plan.	▪ Family or carers visits were adapted on top of taking into consideration Directorate-General of Health guidelines regarding visits and visitors (e.g., building of an adapted wall of plastic which allows hugging)
**Identification and management of ill residents**	▪ Non-compliance with criteria admission for new residents.<br>▪ Insufficient planning to accommodate the necessary travelling of residents to receive specialized care elsewhere.	▪ Activation of an emergency plan to relocate non-infected residents to a closed private hotel facility and manage the health of infected residents inside the nursing home facilities.
**Access to facilities**	▪ Lack of visual cues and information across the institution (including at the entrance).<br>▪ Insufficient planning on how to deal with external service providers that need access to the interior of the facility.	▪ Definition of new processes regarding goods supplies (e.g., orders are placed via e-mail, goods are delivered in a designated outside area, and goods are disinfected prior taken in; invoices are digital instead of paper-based).
