# Supplementary material for: COVID-19 Preparedness and Perceived Safety in Nursing Homes in Southern Portugal: A Cross-Sectional Survey-Based Study in the Initial Phases of the Pandemic
Source: Int J Environ Res Public Health. 2021 Jul 28;18(15):7983. doi: 10.3390/ijerph18157983 (PMC8345424; doi:10.3390/ijerph18157983)
Supplement: Supplementary file 1 [file ijerph-18-07983-s001.zip › File S6.pdf]

Supplementary File 6

Explorative Factor Analysis: factor loadings and communalities, and scale items' reliability

Solutions for two and three factors were each examined using a principal axis factor analysis with varimax and oblimin rotations of the factor loading matrix. A total of two items were excluded (item 4 and 6) given their Kaiser-Meyer-Olkin (KMO) measure of sampling adequacy and having failed to meet the criteria of having a primary factor loading and no cross-loading of 0.3 or above. An oblimin rotation provided the best factor structure and met all relevant assumption criteria (determinant = 0.02; overall KMO = 0.798; KMO for individual items greater than 0.75; Bartlett's sphericity p-value <0.001). Two factors were retained, which combined explained 69% of the variance. Factor 1, which we labelled as *Fear attributed to COVID-19*, was comprised of 4 items that accounted for 43.9% of the variance with factor loading from – 0.047 to 0.916 and a Cronbach's α = 0.838. The other factor was that of *Absenteeism attributed to COVID-19*, which was comprised of 4 items that accounted for 24.8% of the variance and showed a Cronbach's α = 0.854. Overall, after excluding two items for low adequacy sampling, the 8-item scale showed a reliability of 0.811 and a correlation of 0.336 among fear and absenteeism attributed to COVID-19.

Factor loading and communalities based on a principal axis factoring with oblimin rotation (N=599), and scale items' reliability.

Item	Rotated factor loading		Communalities	Reliability	
	1	2		Item-total correlation	Cronbach's α if item deleted
**Factor 1: Fear attributed to COVID-19** (Cronbach's $\alpha_{N=677} = 0.838$)					
Staff have felt fear of becoming infected with the virus that causes COVID-19 because of their job-related tasks	0.794	0.029	0.646	0.731	0.765
Staff have felt fear of infecting nursing home residents or colleagues while at work	0.916	– 0.056	0.807	0.775	0.745
Staff have felt fear of infecting their family members	0.873	– 0.063	0.728	0.751	0.757
Staff have been filled with anxiety because of the use of personal protective equipment at work	0.455	0.075	0.235	0.443	0.888
**Factor 2: Absenteeism attributed to COVID-19** (Cronbach's $\alpha_{N=617} = 0.854$)					
Staff have been missing work out of fear of becoming infected with the virus that causes COVID-19	– 0.014	0.799	0.630	0.709	0.799
Staff have been missing work out of fear of infecting people at their household	– 0.047	0.877	0.744	0.756	0.778
Staff have been missing work because they were recommended to stay at home in quarantine	– 0.026	0.767	0.575	0.706	0.800
Staff have been missing work to provide assistance to family members (e.g. taking care of children)	0.100	0.608	0.421	0.598	0.854
**Eigenvalue**	3.513	1.981			
**% of total variance**	43.9%	24.8%			
**Correlation among factors**	0.336				
**8-item Cronbach's α**	0.811				
