# Supplementary material for: COVID-19 Preparedness and Perceived Safety in Nursing Homes in Southern Portugal: A Cross-Sectional Survey-Based Study in the Initial Phases of the Pandemic
Source: Int J Environ Res Public Health. 2021 Jul 28;18(15):7983. doi: 10.3390/ijerph18157983 (PMC8345424; doi:10.3390/ijerph18157983)
Supplement: Supplementary file 1 [file ijerph-18-07983-s001.zip › File S7.pdf]

Supplementary File 7

Key areas where respondents felt a need for support from others: proportion comparison between Algarve and Alentejo.

Item	Algarve	Alentejo	IRR (95% CI)	p-value
I do not feel a need for support from others	1:4	1:4	1.139 (0.845 – 1.541)	0.380
Dealing with my own negative mood	1:9	1:13	1.433 (0.851 – 2.469)	0.156
Dealing with stress	1:3	1:3	0.833 (0.644 – 1.080)	0.153
Dealing with fear/anxiety	1:4	1:3	0.713 (0.537 – 0.944)	0.014
Dealing with the emotions of others	1:5	1:6	1.053 (0.736 – 1.513)	0.770
Occupying myself with other activities	1:6	1:10	1.527 (0.987 – 2.403)	0.047
Sleeping	1:4	1:4	1.131 (0.833 – 1.541)	0.415
Dealing with grief	1:17	1:27	1.533 (0.732 – 3.381)	0.227
Psychological support	1:67	1:25	0.369 (0.115 – 1.041)	0.035

IRR: Incidence rate ratio. CI: Confidence interval.
