# Supplementary material for: COVID-19 Preparedness and Perceived Safety in Nursing Homes in Southern Portugal: A Cross-Sectional Survey-Based Study in the Initial Phases of the Pandemic
Source: Int J Environ Res Public Health. 2021 Jul 28;18(15):7983. doi: 10.3390/ijerph18157983 (PMC8345424; doi:10.3390/ijerph18157983)
Supplement: Supplementary file 1 [file ijerph-18-07983-s001.zip › File S8.pdf]

# Supplementary File 8

**Algarve:** Nursing home resident safety culture percent positive, negative, and neutral scores (Individual items and composite scores).

| | | Percent **positive** response to item | Percent **negative** response to item | Percent **neutral** response to item | |
|---|---|---|---|---|---|
| | **Teamwork** | **78%** | **6%** | **16%** | Average percent positive/negative/neutral response across the composite items |
| A1 | Staff in this nursing home treat each other with respect | 81% | 4% | 15% | |
| A2 | Staff support one another in this nursing home | 75% | 6% | 18% | |
| A5 | Staff feel like they are part of a team | 80% | 6% | 14% | |
| A9 | When someone gets really busy in this nursing home, other staff help out | 75% | 9% | 16% | |
| | **Staffing** | **61%** | **22%** | **17%** | Average percent positive/negative/neutral response across the composite items |
| A3 | We have enough staff to handle the workload | 55% | 26% | 19% | |
| A8 R | Staff have to hurry because they have too much work to do | 33% | 45% | 22% | |
| A16 | Residents' needs are met during shift | 89% | 2% | 9% | |
| A17 R | It is hard to keep residents safe here because so many staff quit their jobs | 68% | 16% | 16% | |
| | **Compliance with procedures** | **52%** | **29%** | **19%** | Average percent positive/negative/neutral response across the composite items |
| A4 | Staff follow standard procedures to care for residents | 85% | 5% | 10% | |
| A6 R | Staff use shortcuts to get their work done faster | 15% | 59% | 26% | |
| A14 R | To make work easier, staff often ignore procedures | 56% | 24% | 20% | |
| | **Training and skills** | **68%** | **13%** | **19%** | Average percent positive/negative/neutral response across the composite items |
| A7 | Staff get the training they need in this nursing home | 71% | 10% | 20% | |
| A11 | Staff have enough training on how to handle difficult residents | 55% | 22% | 23% | |
| A13 | Staff understand the training they get in this nursing home | 79% | 6% | 15% | |
| | **Nonpunitive response to mistakes** | **55%** | **21%** | **24%** | Average percent positive/negative/neutral response across the composite items |
| A10 R | Staff are blamed when a resident is harmed | 45% | 25% | 30% | |
| A12 R | Staff are afraid to report their mistakes | 47% | 31% | 22% | |
| A15 | Staff are treated fairly when they make | 61% | 14% | 25% | |
| A18 | Staff feel safe reporting their mistakes | 66% | 12% | 22% | |

**Alentejo:** Nursing home resident safety culture percent positive, negative, and neutral scores (Individual items and composite scores).

| | | Percent **positive** response to item | Percent **negative** response to item | Percent **neutral** response to item | |
|---|---|---|---|---|---|
| | **Teamwork** | **79%** | **6%** | **15%** | Average percent positive/negative/neutral response across the composite items |
| A1 | Staff in this nursing home treat each other with respect | 84% | 4% | 12% | |
| A2 | Staff support one another in this nursing home | 78% | 5% | 17% | |
| A5 | Staff feel like they are part of a team | 81% | 6% | 13% | |
| A9 | When someone gets really busy in this nursing home, other staff help out | 72% | 9% | 19% | |
| | **Staffing** | **59%** | **22%** | **19%** | Average percent positive/negative/neutral response across the composite items |
| A3 | We have enough staff to handle the workload | 56% | 24% | 20% | |
| A8 R | Staff have to hurry because they have too much work to do | 29% | 47% | 24% | |
| A16 | Residents' needs are met during shift | 86% | 3% | 11% | |
| A17 R | It is hard to keep residents safe here because so many staff quit their jobs | 67% | 12% | 21% | |
| | **Compliance with procedures** | **49%** | **33%** | **18%** | Average percent positive/negative/neutral response across the composite items |
| A4 | Staff follow standard procedures to care for residents | 85% | 5% | 10% | |
| A6 R | Staff use shortcuts to get their work done faster | 11% | 66% | 23% | |
| A14 R | To make work easier, staff often ignore procedures | 51% | 27% | 22% | |
| | **Training and skills** | **72%** | **10%** | **18%** | Average percent positive/negative/neutral response across the composite items |
| A7 | Staff get the training they need in this nursing home | 76% | 8% | 16% | |
| A11 | Staff have enough training on how to handle difficult residents | 61% | 19% | 20% | |
| A13 | Staff understand the training they get in this nursing home | 78% | 5% | 17% | |
| | **Nonpunitive response to mistakes** | **55%** | **18%** | **27%** | Average percent positive/negative/neutral response across the composite items |
| A10 R | Staff are blamed when a resident is harmed | 50% | 23% | 27% | |
| A12 R | Staff are afraid to report their mistakes | 43% | 30% | 27% | |
| A15 | Staff are treated fairly when they make | 64% | 10% | 26% | |
| A18 | Staff feel safe reporting their mistakes | 63% | 9% | 28% | |
